# Gene profiling of SEC13, SMAD7, GHRL, long non-coding RNA GHRLOS, HIF-1α in gastric cancer patients

**DOI:** 10.1038/s41598-022-10402-w

**Published:** 2022-04-21

**Authors:** Neveen A. Hussein, Mona M. Rashad, Azza S. Abdou, Amr M. Hussein, Hagar M. Mohamed

**Affiliations:** 1 Applied Medical Chemistry Department, Medical Research Institute, Alexandria University, Alexandria, Egypt; 2 Human Physiology Department, Medical Research Institute, Alexandria University, Alexandria, Egypt; 3 Cancer Management and Research Department, Medical Research Institute, Alexandria University, Alexandria, Egypt

**Keywords:** Cancer, Molecular biology, Biomarkers, Oncology

## Abstract

Even with considerable progress in cancer researches, gastric cancer is still one of the global health problems. Recognition of the differential expressed genes in GC is the most appropriate approach for establishing new diagnostic targets. This study evaluates SEC13, SMAD7, GHRL, lncRNA GHRLOS, HIF-1α genes profiling as well as HIF-1α protein level for GC. The expression of selected genes, serum HIF-1α and CEA protein levels were determined for 50 GC patients and 50 healthy controls by real-time RT-PCR, ELISA, and ELICA respectively. The sensitivities of these parameters as diagnostic biomarkers were evaluated. SMAD7, HIF-1α expression, serum HIF-1α, and CEA level were significantly upregulated in GC patients as compared to the control group (*P* = 0.024, < 0.001) and had significant positive correlations between each other except SMAD7 with serum HIF-1α, and CEA level. On the other hand, SEC13, GHRL, and lncRNA GHRLOS expression were significantly downregulated in GC patients (*P* =  < 0.001, 0.025, < 0.001 respectively) and had significant positive correlations with each other (*P* < 0.001). Significant negative correlations were observed between most of both groups. All studied parameters were associated with GC clinical stages except SMAD7 was associated with stage IV only (*P* = 0.005) and GHRL did not associate with tumor stages (*P* ˃ 0.05). All studied parameters may be promising biomarkers for the early diagnosis of GC. SMAD7, HIF-1α gene, and HIF-1α protein may be jointly implicated in cancer development and prognosis, while SEC13, GHRL, and lncRNA GHRLOS may act as tumor suppressors.

## Introduction

Globally, gastric cancer (GC) is still a significant cancer that is responsible for more than one million new cases in 2020 (nearly 1,089,103) and 769,000 deaths (one in every 13 deaths). Moreover, it is 5th for incidence (5.6%) and 4th for mortality (7.7%). The rate of GC in men is two-fold higher than in females. In Egypt, GC is 10th cause of cancer with incidence and mortality rates 3.353 (2.5%) and 2.631 (3.0%) respectively^1^. The high mortality rate of GC may be due to the lack of diagnostic and prognostic markers. Also, the molecular mechanisms responsible for GC are still need realization. So, novel reliable biomarkers are urgently required for early diagnosis. Genes profiling become an attractive focus of research.

Transforming growth factor-β (TGF-β) pathway is multifunctional cytokines which have various impacts on controlling of cell fate throughout embryonic growth and homeostasis of adult tissue. Aberrant TGF-β signaling may cause diseases, including cancer^2^. The canonical TGF-β signaling pathway is through SMAD proteins where its downstream events involve formation of SMAD2 or 3 complexes with SMAD4, translocated to nucleus and subsequent stimulation of target genes. Without ligand, the inhibitory SMADs (SMAD6 and SMAD7) are located mainly in the nucleus. By activation of TGF-β receptor, the inhibitory SMADs accumulate in the cytoplasm and associate with the activated receptor, provoking TGF-β signaling^3^.

SEC13 is a component of the endoplasmic reticulum and the nuclear pore complex (NPC) and is thought to function as scaffold to NPC. Additionally, the nucleoporin SEC13 is associated with chromatin and directly has an important role in transcriptional regulations^4^.

SMADs genes encode transcriptional modulators and signal transducers that mediate several signaling pathways like Wnt/β-catenin, Hippo and TGF-β^5^. SMAD7 gene (small mothers against decapentaplegic) is induced by TGF-β that encodes for negative regulators of TGF-β/SMAD pathway^6^. SMAD7 may antagonize TGF-β signaling in nucleus via interrupting a formation of SMAD-DNA complex^7^. Furthermore, SMAD7 recruits E3 ubiquitin ligases (SMAD ubiquitination regulatory factor 1/2), and thus supports its ubiquitination-mediated proteasomal and/or lysosomal degradation^8^.

Ghrelin gene (GHRL) translates for 28-amino acid peptide hormone (ghrelin) which was initially purified by the stomach mucosa and acts as a ligand for growth hormone secretagogue receptor. Ghrelin is a vital player in different biological processes, involving fat metabolism, growth hormone release, immune system and gut motility^9^.

LncRNA is a group of non-protein coding RNAs exceeding 200 nucleotides and according to its biological positions and roles can categorized into sense, antisense, intergenic, and intronic lncRNA^10^. Most lncRNAs are controlling transcriptional and post-transcriptional levels like mRNA and protein stability, gene splicing, and nuclear cytoplasm exporting levels^11^. GHRLOS (Ghrelin Gene Opposite Strand) is the natural antisense transcript and exhibit numerous unique characteristics of non-coding genes as extensive splicing, 5′ capping, short open reading frames, and polyadenylation^12^.

Hypoxia-inducible factors (HIF-1α, -2α, -3α, -1β) are critical transcriptional regulator of cell response to hypoxia^13^ where they involved in immunity, energy metabolism, microbial homeostasis and renewal^14^. HIF-1α is a prospective target gene implicated in bioenergetic metabolism including upregulate glucose transporters (GLUT1, GLUT4 and GLUT8), the genes expression of glycolysis enzyme^15^, and lactate dehydrogenase-5 (LDH-5). The produced lactate by LDH is circulated, acidifying the cellular matrix and could additional prompt aggressive behavior^16^.

Therefore, this study aimed to evaluate the expression of SEC13, SMAD7, GHRL, lncRNA GHRLOS, HIF-1α genes as well as HIF-1α protein level as diagnostic biomarkers for GC. Correlations of these parameters with clinicopathological characteristics of GC patients were also estimated.

## Subjects and methods

The study was conducted on 50 newly diagnosed GC patients (age range, 44–69 years; median 50 years) who were selected from those admitted to Medical Research Institute, Cancer Management and Research Department, Alexandria University and 50 healthy volunteers (control group, age range, 40–65 years; median 48 years).

Patients underwent gastroscopy and diagnosed pathologically GC were included in this study while patients who received surgical resection or adjuvant chemotherapy and/or radiotherapy or had a history of other malignant disease were excluded.

The study has been approved by the Ethics Committee of Medical Research Institute, Alexandria University and according with The Code of Ethics of the World Medical Association (Declaration of Helsinki) for human study. Assigned informed consent was obtained from all the participants.

### Blood sampling

Fasting blood (6 ml) was withdrawn from each participant, patients before surgery and controls. In K_3_EDTA-containing tube, 3 ml blood was pipetted for quantification of SEC13, SMAD7, GHRL, lncRNA GHRLOS, and HIF-1α genes by real time PCR. The remaining blood (3 ml) was collected in tube without anticoagulant, kept at room temperature and then centrifuged 10 min at 6000 rpm. Sera have been utilized for quantification of HIF-1α and CEA protein by ELISA and ELICA respectively.

### Relative quantification of SEC13, SMAD7, GHRL, lncRNA GHRLOS, and HIF-1α genes

Total RNA was extracted and purified by QIAamp RNA Mini kit (Qiagen, USA). For each sample, the absorbance at 260 nm and A260/A280 ratio were measured to assess the RNA concentration and purity respectively (NanoDrop spectrophotometer, Thermo Fisher Scientific 2000, USA). High-Capacity cDNA Reverse Transcription Kits were used to synthesize cDNA (Applied Biosystems, USA).

#### Real time PCR

Amplification reaction of these genes was performed in 25 µl using SYBR Green Master Mix containing dNTPs, buffer, SYBR® Green dye, and thermostable hot-start DNA polymerase (Thermo Fisher Scientific Inc). The PCR conditions were 95 °C for 15 min followed by 40 cycles at 94 °C for 15 s and 70 °C for 60 s. The genes relative quantification was normalized to GAPDH (endogenous control) and calculated using 2^−∆∆CT^ method.

SEC13-Hs01115007_m1, #4448892 (Invitrogen- Life Technologies-Thermo Fisher Scientific Inc, USA).

SMAD7: forward: 5′-CCTGCCATTGTAGCGTCTTTC-3′ 
  reverse: 5′-CCCTTGGGAAGCCCATCT-3′
GHRL: forward: 5′ GGGCAGAGGATGAACTGGAA-3′ 
  reverse: 5′-CCTGGCTGTGCTGCTGGTA-3′
LncRNA GHRLOS: forward: 5′-GGAAACTCCCCTAGCCACA-3′ 
  reverse: 5′-GCATCTCTCCTCTGTTCCGT-3′
HIF-1α: forward: 5′-TAGCCGAGGAAGAACTATGAAC-3′
  reverse: 5′-CTGAGGTTGGTTACTGTTGGTA-3′
GAPDH: forward: 5′-ATCCTGGGCTACACTGAGCACC-3′ 
  reverse: 5′-AAGTGGTCGTTGAGGGCAATGC-3′

### Serum HIF-1α and CEA

The HIF-1α protein (ng/ml) was measured by ELISA kit (Monobind Inc, USA). The concentration of each sample was calculated from the standard curve (500 ng/ml). The CEA level (ng/ml) was performed by electro chemiluminescence immunoassay (ELICA, Roche Diagnostics Gmbh, Germany) using Cobase 411 immunoassay analyzer.

### Statistical analysis

Data were analyzed by utilizing the IBM SPSS version 20.0. Significance at *P* ˂ 0.05. Chi-square test, for categorical variables, to compare between different groups. The abnormally distributed quantitative variables, Kruskal Wallis and Mann Whitney tests were used. For normally distribution, comparing between two studied groups was done by Student t-test. Spearman coefficient was used for correlation. Receiver operating characteristic curve (ROC) was applied to compare the diagnostic values of studied parameters depending upon the area under the curve (AUC). Elevated AUC relates to a better diagnostic test.

## Results

### Clinicopathological characteristics

Table 1 revealed that 4 patients were less than 50 years while 46 were above 50 years. For sex, 23 were females, while 27 were males. According to staging of GC patients, 5 were of stage II, 17 were of stage III, and 28 were of stage IV. For histological grading, 13 patients had grade II and 37 had grade III. For tumor size, 33 patients were > 5 cm and 17 were < 5 cm. Concerning CEA level, 9 patients had normal level while 41 had elevated level. Regarding the metastatic state, 30 of them had metastasis to liver while 20 did not. Depending on the family history, 35 of them had a family history while 15 did not. Finally, with respect to the pathological Lauren classification, 5 patients were intestinal while 45 of them were diffused type.

**Table 1:** Clinicopathological characteristics of gastric cancer patients.

	Gastric cancer patients (n = 50)	
	No	%
**Age**		
< 50	4	8.0
≥ 50	46	92.0
**Sex**		
Female	23	46
Male	27	54
**Stage**		
II	5	10.0
III	17	34.0
IV	28	56.0
**Grade**		
II	13	26.0
III	37	74.0
**Tumor size**		
< 5	17	34.0
> 5	33	66.0
**CEA**		
Normal	9	18.0
Elevated	41	82.0
**Metastatic state**		
No	20	40.0
Yes	30	60.0
**Family history**		
No	15	30.0
Yes	35	70.0
**Lauren classification**		
Intestinal	5	10.0
Diffused	45	90.0

*n*: number of cases.

### Molecular and biochemical parameters

Statistical analysis of these results revealed that SMAD7, HIF-1α expression, serum HIF-1α and CEA level were significantly upregulated in GC patients as compared to control group (*P* = 0.024, < 0.001, < 0.001, < 0.001) and had significant positive correlations between each other except SMAD7 with serum HIF-1α (r_s_ = 0.177, *P* = 0.078), and CEA level (r_s_ = 0.169, *P* = 0.093). On the other hand, SEC13, GHRL and lncRNA GHRLOS expression were significantly downregulated in GC patients as compared to control group (*P* =  < 0.001, 0.025, < 0.001 respectively) and had significant positive correlations with each other (r_s_ = 0.378, 0.672, 0.397 respectively, *P* < 0.001). Significant negative correlations were observed between most of both groups (r_s_ = − 0.2, *P* = 0.046) (r_s_ = − 0.258, *P* = 0.015) (r_s_ = − 0.254, *P* = 0.011) (r_s_ = − 0.248, *P* = 0.013 ) (r_s_ = − 0.418, r_s_ = − 0.644, r_s_ = − 0.473, r_s_ = − 0.715, *P* =  < 0.001) (Tables 2, 3).

**Table 2:** SEC13, GHRL, lncRNA GHRLOS, SMAD7, HIF-1α genes expression, serum HIF-1α (ng/ml) and CEA level (ng/ml) in control and gastric cancer patients' groups.

Control (n = 50)	Gastric cancer patients (n = 50)	*P*
**SEC13 gene**		
4.50 ± 12.12	0.34 ± 0.42	< 0.001*
0.72 (0.28–2.7)	0.18 (0.12–0.33)	
**GHRL gene**		
4.87 ± 7.48	1.32 ± 3.40	0.025*
0.51 (0.20–6.0)	0.34 (0.11–0.78)	
**LncRNA GHRLOS**		
3.28 ± 7.74	0.11 ± 0.52	< 0.001*
0.88 (0.49–1.60)	0.17 (0.09–0.78)	
**SMAD7 gene**		
4.48 ± 8.60	5.36 ± 12.04	0.024*
0.56 (0.28–3.6)	1.56 (0.59–5.20)	
**HIF-1α gene**		
3.81 ± 6.56	5.60 ± 6.40	< 0.001*
0.65 (0.24–5.39)	4.81 (1.37–7.21)	
**HIF-1α protein**		
17.64 ± 4.12	191.6 ± 43.94	< 0.001*
19.10 (16.60–20.40)	198.7 (167.0–224.2)	
**CEA protein**		
2.93 ± 1.01	71.77 ± 45.91	< 0.001*
3.02 (2.20–3.60)	84.50 (37.0–97.0)	

Mean ± S.D. and Median (IQR).

*n:* number of cases.

*P: P* value for comparing between GC patients and control group utilizing Mann Whitney test.

*Significance at *P* ≤ 0.05.

**Table 3:** Correlation between SEC13, GHRL, lncRNA GHRLOS, SMAD7, HIF-1α genes expression, serum HIF-1α and CEA levels in gastric cancer patients' group.

	GHRL	LncRNA GHRLOS	SMAD7	HIF-1α	CEA protein	HIF-1α protein
**SEC13**						
r_s_	0.378	0.672	− 0.200	− 0.258	− 0.418	− 0.473
*P*	< 0.001*	< 0.001*	0.046*	0.015*	< 0.001*	< 0.001*
**GHRL**						
r_s_		0.397	0.162	− 0.254	− 0.147	− 0.248
*P*		< 0.001*	0.107	0.011*	0.144	0.013*
**LncRNA GHRLOS**						
r_s_			− 0.146	− 0.180	− 0.644	− 0.715
*P*			0.147	0.074	< 0.001*	< 0.001*
**SMAD7**						
r_s_				0.710	0.169	0.177
*P*				< 0.001*	0.093	0.078
**HIF-1α**						
r_s_					0.329	0.349
*P*					< 0.001*	< 0.001*
**CEA protein**						
r_s_						0.665
*P*						< 0.001*

r_s_: Spearman coefficient.

**Figure 1:**
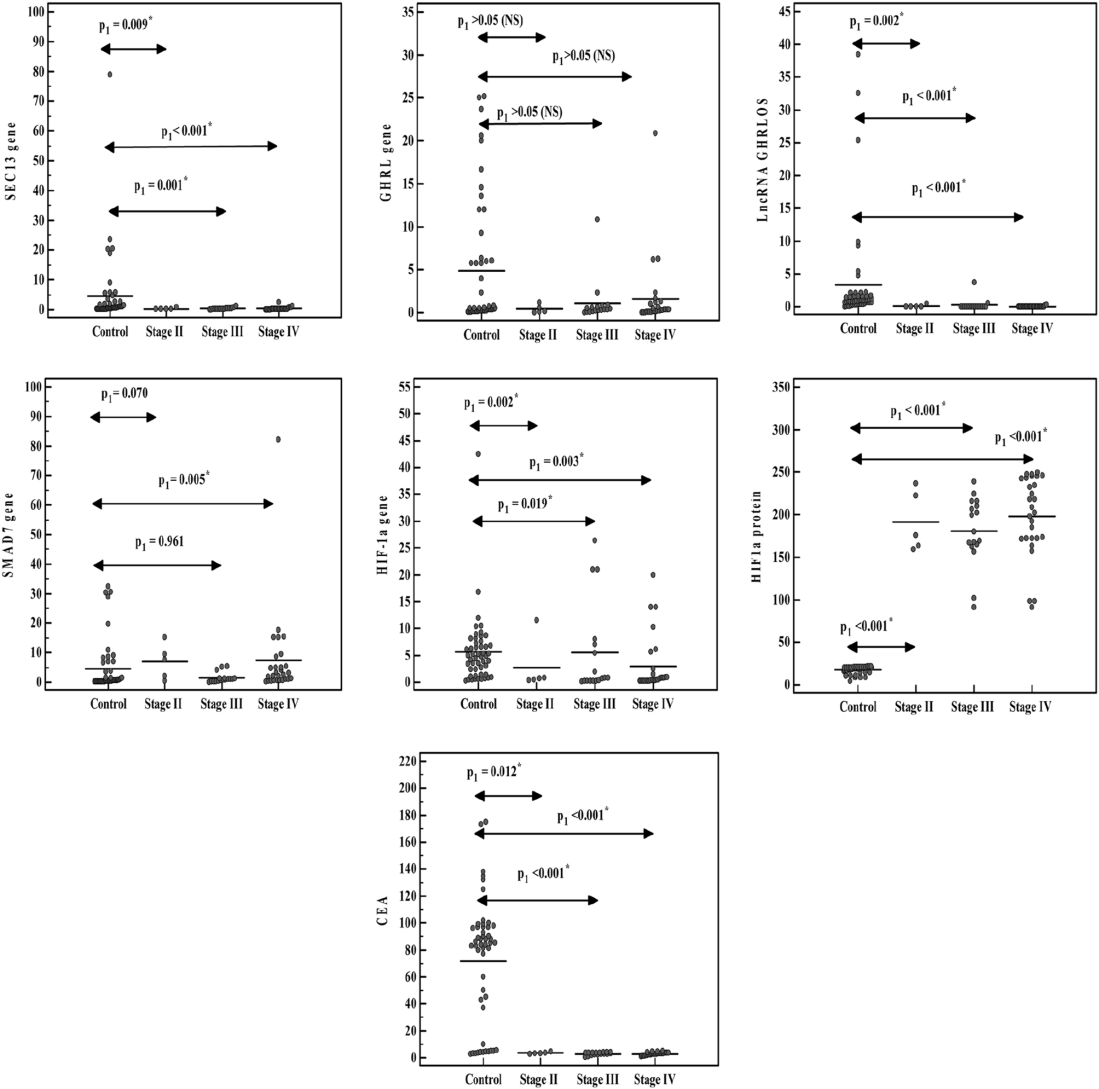
SEC13, GHRL, lncRNA GHRLOS, SMAD7, HIF-1α expression, serum HIF-1α (ng/ml) and CEA levels (ng/ml) in control and gastric cancer patient groups with different stage.

All studied parameters were associated with GC clinical stages, as their relative median values upregulated with stages (II, III, IV) except SMAD7 was associated with stage IV only and GHRL did not associate with tumor stages (Table 4, Fig. 1).

**Table 4:** SEC13, GHRL, lncRNA GHRLOS, SMAD7, HIF-1α genes expression, serum HIF-1α and CEA levels in control and gastric cancer patients' groups with different stages.

Control (n = 50)	Gastric cancer patients (n = 50)			*p*
	Stage II (n = 5)	Stage III (n = 17)	Stage IV (n = 28)	
**SEC13**				
4.50 ± 12.12	0.28 ± 0.32*	0.33 ± 0.30*	0.35 ± 0.50*	< 0.001*
0.72	0.14	0.18	0.27	
**GHRL**				
4.87 ± 7.48	0.40 ± 0.47	1.10 ± 2.55	1.61 ± 4.10	0.132
0.51	0.15	0.32	0.39	
**LncRNA GHRLOS**				
3.28 ± 7.74	0.11 ± 0.21*	0.26 ± 0.88*	0.03 ± 0.06*	< 0.001*
0.88	0.09	0.15	0.02	
**SMAD7**				
4.48 ± 8.60	6.93 ± 5.98	1.47 ± 1.71	7.45 ± 15.53*	0.013*
0.56	5.72	0.95	7.57	
**HIF-1α**				
3.81 ± 6.56	3.76 ± 3.33*	4.61 ± 4.35*	13.65 ± 16.17*	0.001*
0.65	3.13	3.46	6.73	
**HIF-1α protein**				
17.64 ± 4.12	191.52 ± 35.47*	180.85 ± 40.32*	198.14 ± 47.31*	< 0.001*
19.1	175.6	179.8	205.5	
**CEA protein**				
2.93 ± 1.01	49.28 ± 47.22*	70.22 ± 44.48*	73.09 ± 47.06*	< 0.001*
3.02	45	85	85	

*P: P* value for comparing between all groups utilizing Kruskal Wallis test, pairwise comparison between each two groups was performed by Post Hoc Test.

*Significance at *P* ≤ 0.05.

There were no correlations between all studied parameters and clinicopathological characteristics (age, grade, tumor size, metastatic state, family history and Lauren classification) of GC patients except a significant positive correlation of SMAD7 with tumor size and metastatic state (*P* = 0.008, 0.007 respectively) and a significant negative correlation of SEC13 with family history (*P* = 0.033).

**Figure 2:**
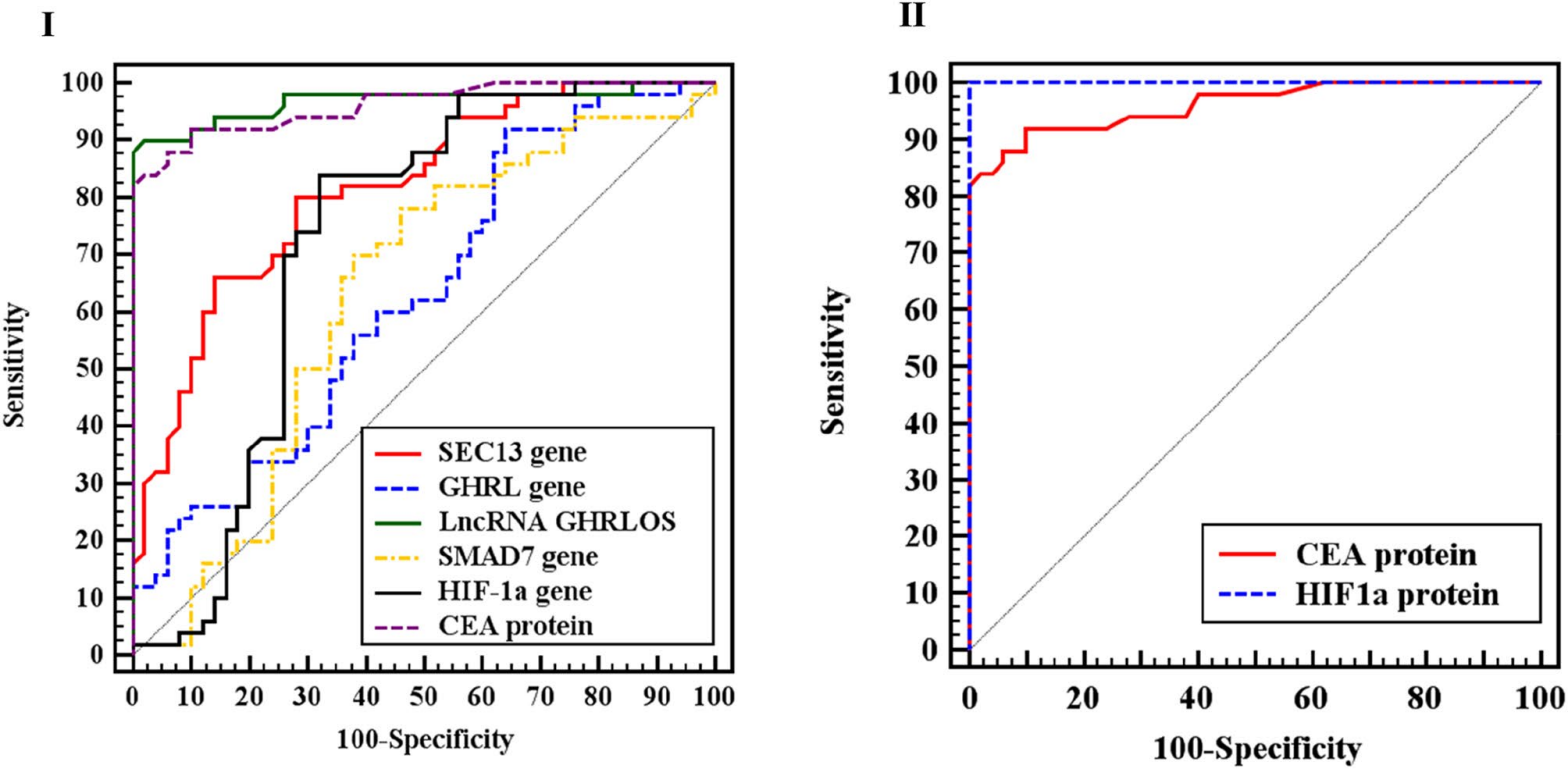
ROC curve for (I) SEC13, GHRL, lncRNA GHRLOS, SMAD7, HIF-1α genes and serum CEA, (II) serum HIF-1α and CEA.

As shown in Table 5 Fig. 2; AUC values were significant high for all parameters SEC13, SMAD7, GHRL, lncRNA GHRLOS, HIF-1α genes, HIF-1α and CEA protein (81.6%, 63.1%, 63%, 96.8%, 72.4%, 100%, 96.1% respectively). SEC13 had 80% sensitivity, 72% specificity, and cut-off ≤ 0.389. SMAD7 had 70% sensitivity, 62% specificity and cut-off > 0.752. GHRL had 60% sensitivity, 58% specificity, and cut-off ≤ 0.39. LncRNA GHRLOS had 90% sensitivity, 98% specificity, and cut-off ≤ 0.03. HIF-1α expression had 84% sensitivity, 68% specificity, and cut-off > 0.895. Serum HIF-1α protein had 100% sensitivity, 100% specificity, and cut-off > 2.18. Finally, serum CEA had 92% sensitivity, 90% specificity, and cut-off > 3.98.

**Table 5:** Diagnostic accuracy of SEC13, GHRL, lncRNA GHRLOS, SMAD7, HIF-1α genes expression, serum HIF-1α and CEA levels.

	AUC	Cut off^#^	Sensitivity	Specificity	95% CI	PPV	NPV	Accuracy	*P*
SEC13	0.816	≤ 0.389	80.0	72.0	0.734 –0.898	74.1	78.3	76.0	< 0.001*
GHRL	0.630	≤ 0.390	60.0	58.0	0.521 –0.739	58.8	59.2	59.0	0.026*
LncRNA GHRLOS	0.968	≤ 0.030	90.0	98.0	0.930 –1.000	97.8	90.7	94.0	< 0.001*
SMAD7	0.631	> 0.752	70.0	62.0	0.518 –0.743	64.8	67.4	66.0	0.024*
HIF-1α	0.724	> 0.895	84.0	68.0	0.617 –0.831	72.4	81.0	76.0	< 0.001*
HIF-1α protein	1.000	> 2.180	100.0	100.0	1.000 –1.000	100.0	100.0	100.0	< 0.001*
CEA protein	0.961	> 3.980	92.0	90.0	0.926 –0.996	90.2	91.8	91.0	< 0.001*

*CI:* Confidence intervals, *AUC:* area under the curve, *PPV:* positive predictive value, *NPV:* negative predictive value.

*Statistically significant at *P* ≤ 0.05.

^#^Cut off was choose according to Youden index.

## Discussion

Even with considerable progress in cancer researches, GC is still one of the global health problems. Genes profiling is the most appropriate approaches for establishing new diagnostic biomarkers.

The present results revealed significant down regulation of SEC13 and up regulation of SMAD7 expression in GC patients group as compared to control group. In normal and premalignant cells, TGF-β mainly functions as a tumor suppressor through promoting apoptosis, maintaining genome stability, and reducing proliferation. While cancer cells could evade TGF-β suppression effect; they use TGF-β advertising functions to acquire a growth benefit and undergo processes, like epithelial-mesenchymal transition, which facilitate their invasion, and migration^17^.

Under well oxygenated conditions, SMAD7 is an effective inhibitor of cancer invasion. Hypoxia, which is commonly met in solid tumors, activates SMAD7 expression in HIF- and von Hippel-Lindau protein-dependent manner. The upregulated SMAD7 expression in tumors is correlated with hypoxic gene expression where hypoxia might convert SMAD7 from invasion inhibitor to activator^18^. Hypoxia triggers posttranslational covalent protein alterations (hydroxylation, and phosphorylation). Provoking or preventing these alterations, hypoxia might favor further formation of permanent R-SMAD/SMAD7 complexes and SMAD7 could dephosphorylate R-SMADs then, impact SMAD-stimulated transcription. Moreover, HIF is well-known to bind SMAD3 and changes the activation of hypoxic gene^19^. On the contrary, it would be reasonable that HIF might modify R-SMAD-SMAD4-SMAD7 complex binding to DNA. Additionally, hypoxia has been recognized to stimulate a great number of genes, so hypoxia-caused alterations in gene expression might change responses of TGF-β to support instead of prevent invasion^18^.

SMADs access to their genes is exactly signal dependent, causing SMAD nuclear translocation an important step in TGF-β signal transduction into nucleus. SEC13 has specific properties for SMADs, where SEC13 can provide mechanism by which TGF-β accelerates nuclear import rate of SMADs. Furthermore, SEC13 participates in directing SMADs to their chromatin binding sites and contributes to SMAD-facilitated transcriptional control^20^. According to SEC13 functions, the significant down regulation of SEC13 expression in GC patients may be one of the factors that leads to TGF-β signaling aberrant. This study confirmed decrease in SEC13 expression with all cancer stages (II, III, IV); while SMAD7 tended to be especially highly expressed in GC patients with stage IV, which could explain the significant negative correlation between SEC13 and SMAD7.

The present study revealed down expression of GHRL in GC patients as compared to control group. Also, GHRL expression was not associated with GC stages. This result agreed with Pritchett et al., 2020 and Hu et al., 2021^21,22^. Ghrelin has anti-inflammatory effects where it alleviates production of nuclear factor-κB and pro-inflammatory cytokines. Additionally, GHRL suppress macrophage-produced inflammatory cytokines and cyclooxygenase-2 expression^23^. GHRL activates gastric vagus nerve which delivers the gastrointestinal tract immune information to the hypothalamus^24^. Therefore, ghrelin's anti-inflammatory impacts may protect against tumorigenesis^25^.

AMP-activated protein kinase (AMPK) is the master sensor of cell energy level and it has been proven to control the cell critical functions, involving growth and metabolism, and constantly could be implicated in initiation and development of tumor^26^. Also, AMPK is a negative regulator of Warburg effect to suppress of GC development^27^. A recent study by Hu et al. 2021^22^ recognized that overexpression of GHRL may inhibit GC cell proliferation, invasion, and support apoptosis by activating AMPK pathway. GHRL knockdown enhanced uptake of glucose and release of lactic acid, indicating that GHRL provoked the anti-Warburg effect through AMPK signaling pathway to prevent GC. Accordingly, the observed decrease in GHRL expression in GC patients led to assume that GHRL may function as the tumor suppressor.

The present results showed a significant down regulation in lncRNA GHRLOS in GC patients group as compared to control group. As well as a significant association with GC stages (II, III, IV). These findings are consistent with other research which proved that lncRNA GHRLOS may act as a tumor suppressor during colorectal carcinogenesis and its down regulation stimulates CRC progression^28^ through its functional and regulatory roles in ghrelin axis^12^.

LncRNA GHRLOS has been recognized as an overlapping gene on GHRL antisense strand and can serve as a suppressor of its overlapping gene^28^. Interestingly, it has been proven that ghrelin-AMPK signaling utilizes the anti-Warburg effect to inhibit GC progress^22^. Regarding lncRNA GHRLOS, a previous study suggested that lncRNA GHRLOS can play role in the regulation of gluco-metabolism^29^. Therefore, it can be suggested that lncRNA GHRLOS has comparable inhibitory effect on cancer cell as it is related to GHRL gene.

In accordance with previous studies^30,31^, the present results revealed that HIF-1α gene expression and protein level were significantly elevated in GC patient as compared to control group and were significantly associated with GC clinical stages (II, III, IV). Furthermore, the level of HIF-1α protein was in consistent with its mRNA expression in GC patient.

In cancer cells the main feature in response to hypoxia is induction of HIF-1α as well as its downstream target to enhance blood vessel formation, and aggression^32^. Reactive oxygen species (ROS) induce HIF-1α transcriptional activity through stimulation of NF-κB, extracellular signal-regulated kinases (ERK1/2) and PI3K/Akt/m-TOR pathways. Reactive nitrogen species (RNS) induce S-nitrosation of prolyl 4-hydroxylase 2 (PHD2) to elevate HIF-1α stability and activity^14,33^. In addition to oxygen, HIF-1 may be regulated by other stimuli involving hormones (insulin), growth factors (platelet-derived growth factor) and vasoactive peptide (angiotensin-2)^34^.

The present study demonstrated that a significant elevation in CEA in GC patients that trends to be significantly increased with GC clinical stages as compared to control group. The secretion and transcription of CEA are controlled by TGF-β pathway and SMAD3-facilitated tumor growth factor^35^.

Hypoxia increases CEA protein level and its promoter activity directly through HIF-1α binding^36^ and indirectly through increase cellular pH by activation of plasma membrane Na + /H + exchanger^37^. Previous study has revealed that E-box represents an important site for the total activity of CEA promoter and contain potential binding sites for various transcription factors as HIF-1α^38^.

The correlations of all studied parameters with clinicopathological characteristics of GC patients showed some variation than other previous studies^39,40^ and this may be due to difference in sample numbers and methods used.

The observed correlations between all studied parameters are reasoning since under hypoxia condition TGF-β/SMAD signaling is promoted. TGF-β upregulates HIF-1α expression and induces HIF-1α DNA binding activity. TGF-β affects HIF-1α activity and accumulation via enhancing stability of HIF-1α protein^41^. Besides, HIF-1α plays a main role for the elevation of CEA protein level and promotes its activity^36^. Furthermore, SMAD7 is activated by hypoxia in HIF- and VHL-dependent manner and its inhibitory effect on invasion is totally lost^18^. Also, with hypoxic condition, GHRL reduces the level of HIF-1α^42^. Additionally, lncRNAs can act as a direct or indirect regulator of HIFs and can improve or reduce its function in cancer^43^. LncRNA GHRLOS is positively correlated with GHRL and negatively correlated with HIF-1α protein. So that, lncRNA GHRLOS may exert the same inhibitory effect.

The present results indicated that the validity of using SEC13, SMAD7, GHRL, lncRNA GHRLOS, HIF-1α genes, and HIF-1α protein as diagnostic markers for GC. Additionally, HIF-1α protein was superior to lncRNA GHRLOS followed by CEA protein, SEC13, HIF-1α, SMAD7, and GHRL genes for GC diagnosis.

Conclusion: SEC13, SMAD7, GHRL, lncRNA GHRLOS, HIF-1α genes, and HIF-1α protein may be considered as promising biomarkers for the early detection of gastric cancer. SMAD7, HIF-1α gene and HIF-1α protein may be jointly involved in tumor development and prognosis (act as oncogenic factors), while SEC13, GHRL, and lncRNA GHRLOS may act as tumor suppressor factors and thus could be considered as novel therapeutic targets for gastric cancer.

## Data Availability

All data generated or analyzed during this study are included in this published article.
